# Short and Long-Term Effects of hVEGF-A_165_ in Cre-Activated Transgenic Mice

**DOI:** 10.1371/journal.pone.0000013

**Published:** 2006-12-20

**Authors:** Pia Leppänen, Ivana Kholová, Anssi J. Mähönen, Kari Airenne, Suvi Koota, Hannu Mansukoski, Johanna Närväinen, Maria Wirzenius, Leena Alhonen, Juhani Jänne, Kari Alitalo, Seppo Ylä-Herttuala

**Affiliations:** 1 Department of Biotechnology and Molecular Medicine, A.I. Virtanen Institute, University of Kuopio Kuopio, Finland; 2 Technical University of Dresden Dresden, Germany; 3 Faculty of Life Sciences, University of Manchester Manchester, United Kingdom; 4 Molecular/Cancer Biology Laboratory, Biomedicum, University of Helsinki Helsinki, Finland; 5 Department of Medicine and the Gene Therapy Unit, Kuopio University Hospital Kuopio, Finland; Ordway Research Institute Inc., United States of America

## Abstract

We have generated a transgenic mouse where hVEGF-A_165_ expression has been silenced with loxP-STOP fragment, and we used this model to study the effects of hVEGF-A_165_ over-expression in mice after systemic adenovirus mediated Cre-gene transfer. Unlike previous conventional transgenic models, this model leads to the expression of hVEGF-A_165_ in only a low number of cells in the target tissues in adult mice. Levels of hVEGF-A_165_ expression were moderate and morphological changes were found mainly in the liver, showing typical signs of active angiogenesis. Most mice were healthy without any major consequences up to 18 months after the activation of hVEGF-A_165_ expression. However, one mouse with a high plasma hVEGF-A_165_ level died spontaneously because of bleeding into abdominal cavity and having liver hemangioma, haemorrhagic paratubarian cystic lesions and spleen peliosis. Also, two mice developed malignant tumors (hepatocellular carcinoma and lung adenocarcinoma), which were not seen in control mice. We conclude that long-term uncontrolled hVEGF-A_165_ expression in only a limited number of target cells in adult mice can be associated with pathological changes, including possible formation of malignant tumors and uncontrolled bleeding in target tissues. These findings have implications for the design of long-term clinical trials using hVEGF-A_165_ gene and protein.

## Introduction

Vascular endothelial growth factor (VEGF-A) was the first identified member of the family that now includes placental growth factor (PIGF) and several other VEGFs (VEGF-B,-C,-D,-E) [1], [2]. Since then the VEGF family has been shown to play a major role in vascular permeability, angiogenesis and lymphangiogenesis both during embryonic development and in adults. VEGFs have also been used in clinical applications as recombinant proteins or gene therapy [3], [4]. However, long term effects of moderate VEGF over-expression in adults have not been characterized in the context of recombinant protein or gene therapy.

Due to the fundamental requirement of VEGF-A in embryonal development it has been impossible to create viable knockout models for VEGF-A. Even by knockout of a single VEGF-A allele mice were unable to survive [5]–[7]. Also, mice expressing only VEGF-A_120_ but no longer isoforms die within two weeks after birth because of the cardiac failure [8]. VEGF-C knockout mice die due to the lack of lymphatic vessels, while VEGF-C+/− mice survive despite of the defects in lymphatic vessels [9.10]. In contrast to VEGF-A and VEGF-C knockout mice VEGF-B, VEGF-D and PIGF deficient mice are viable, and PIGF and VEGF-B double-knockout mice showed no significant vascular phenotype [11]–[13]. Several transgenic mouse models have been generated using the members of the VEGF family. Transgenic VEGF-A has been expressed in the skin, eyes, lungs, heart or liver under tissue specific promoters [14]–[18]. Also, PIGF transgenic mice have been described [19]. Similar results have been reported from these models, showing that all mice have increased vascularization and vascular permeability. In contrast, studies with transgenic mice over-expressing VEGF-C and VEGF-D under keratinocyte or pancreas specific promoters have demonstrated the role of these growth factors mainly in lymphangiogenesis [20]–[22]. However, in all these models VEGFs are typically expressed in every cell of the target tissue and the models may not fully predict outcome after gene therapy applications where transduction efficiency is typically <1–5% of the cells in target organ [23], [24].

We have generated a transgenic mouse model containing in its genome a loxP-STOP inactivated hVEGF-A_165_ expression cassette, which can be activated by Cre gene transfer in any tissue at any point during life [25]. The main reason for the generation of the current transgenic model was to evaluate potential long-term side effects of therapeutic hVEGF-A_165_ gene transfer. In this construct we have used the same type of enhancer as we have used in our previous gene transfer studies, but employed a more methylation-resistant promoter in order to achieve a long-term expression (CMV immediate early enhancer and the chicken β–actin promoter) [23], [26]. In the present study we have used this mouse model to study short and long-term effects of hVEGF-A_165_ over-expression on the pathology of liver and various other tissues. Systemic AdCre gene transfer was chosen for the study since this leads primarily to the activation of hVEGF-A_165_ in the liver, which is the most common site of unintended biodistribution of adenoviruses after in vivo gene transfer [27], [28].

## Results and Discussion

### Testing of the hVEGF-A_165_ expression cassette and Short-term effects

**Figure 1:**
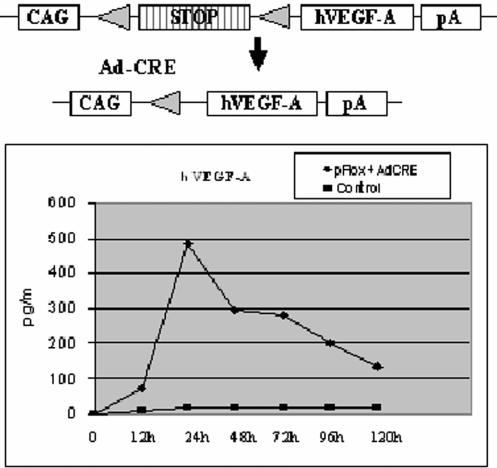
Shematic representation of pFlox hVEGF-A_165_ cassette, where VEGF expression is activated by Cre-mediated excision of STOP cassette and expression of human hVEGF-A_165_ in vitro after AdCre mediated transduction of NIH-3T3 cells with plasmid loxP-STOP-hVEGF-A_165_. CAG = CMV-IE enhancer+chicken β-actin promoter; triangle = loxP, a 34 bp long recombination sequence; STOP = DNA element to prevent VEGF expression; hVEGF-A = human VEGF-A_165_ cDNA; pA = rabbit β-globin polyadenylation signal.

**Figure 2:**
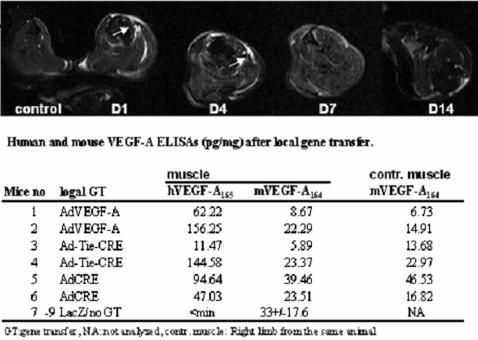
Edema after AdCre mediated local gene transfer detected by MRI and results from human and mouse ELISA analyses after local GT. Discrete edema (white areas pointed with arrows) is clearly visible between day 1 and day 7 under the skin in T2 weighted MRI images in the transduced leg muscles, their fascias, and in the fat tissue within the transduced muscles. ELISA assays were done as described in the Methods.

**Figure 3:**
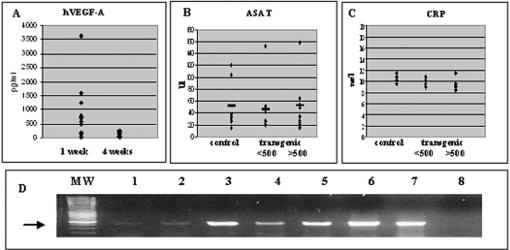
Expression of human and mouse VEGF-A after AdCre gene transfer in vivo. A. hVEGF-A_165_ ELISA from sera after AdCre gene transfer. B. ASAT values in transgenic and control mice after AdCre gene transfer. C. CRP values in transgenic and control mice after AdCre gene transfer. D. Expression of hVEGF-A_165_ mRNA using RT-PCR. Lanes; 1 = Aorta, 2 = pancreas, 3 = kidney, 4 = heart, 5 = spleen, 6 = liver, 7 = positive control and 8 = liver without RT (500 bp pointed with an arrow). <500 and >500 in B and C refer to hVEGF-A_165_ serum levels in two subgroups of transgenic mice.

Before creating transgenic mice the pFlox construct (Figure 1) was tested in vitro and the cassette was found functional causing a strong hVEGF-A_165_ expression after AdCre-mediated excision of the STOP fragment. AdCre and pFlox treated cells expressed hVEGF-A_165_ efficiently reaching the peak expression at 24 h after the transduction. Cells transduced with pFlox alone or hVEGF-A_165_ transgenic mice without gene transfer did not express any detectable amounts of hVEGF-A_165_ during the follow-up time. The functionality of the hVEGF-A_165_ transgene in vivo was verified by MRI, where typical changes were seen as discrete edema localized under the skin in all transduced muscles and their fascia and also in the fat tissue due to increased vessel permeability (Figure 2 and Table 1A). We used ELISA to measure the levels of hVEGF-A_165_ in order to see total protein levels of the transgene both in sera and tissues. The possible gross-reactivity was tested after local gene transfer and no correlations were found between mouse endogenous VEGF-A_164_ and hVEGF-A_165_ levels in the control and the AdCre transduced transgenic mice (Figure 2). Systemic AdCre gene transfer via tail vein was done to 31 transgenic mice and 10 control mice at the age of 2 months. Also, 10 additional transgenic mice were injected with saline or AdLacZ. One week after activation a wide range of hVEGF-A_165_ concentrations were detected in sera of the transgenic mice. We estimate that only 0.1 to 5% of all cells in the liver were transduced after the AdCre gene transfer and the levels of hVEGF-A_165_ were only moderate in comparison to the transgene expression levels achieved in tissues in previously generated transgenic VEGF-A models where all cells in a given tissue express the transgene. For example Dor et al [18] were able to achieve 7- to 11-fold increases in hVEGF-A_165_ expression as compared with the level of endogenous mVEGF-A_164_ After 1 week the levels of hVEGF-A_165_ in serum were ∼50% of the levels achieved after direct gene transfer with adenoviruses encoding hVEGF-A_165_ [26]. During the next 3–4 weeks (Figure 3A) serum hVEGF-A_165_ levels decreased. This could be due to the immune response against adenovirus or toxicity of Cre, even if no signs of pathological responses were found in clinical chemistry or histological sections; apoptotic cells were present in all livers transduced with AdCre, but also in the control mice (data not shown) and no major abnormalities were seen (ASAT and CRP) between the groups (Figure 3B and C). The promoter area was selected to be more resistant to methylation (the chicken β–actin promoter) and in contrast to serum levels, low expression of hVEGF-A_165_ was present in many tissues even after 18 months as analyzed with ELISA ([Table pone-0000013-t001] Table 1) and RT-PCR (Figure 3D).

**Table 1 pone-0000013-t001:**
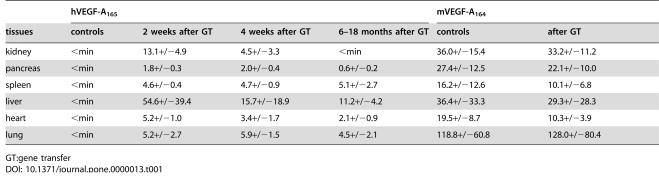
A. Human and mouse VEGF-A ELISAs (pmol/mg) from tissue samples after systemic AdCre gene transfer (mean+/−SD)

	hVEGF-A_165_			mVEGF-A_164_	
tissues	controls	2 weeks after GT	4 weeks after GT	6–18 months after GT	controls	after GT
kidney	<min	13.1+/−4.9	4.5+/−3.3	<min	36.0+/−15.4	33.2+/−11.2
pancreas	<min	1.8+/−0.3	2.0+/−0.4	0.6+/−0.2	27.4+/−12.5	22.1+/−10.0
spleen	<min	4.6+/−0.4	4.7+/−0.9	5.1+/−2.7	16.2+/−12.6	10.1+/−6.8
liver	<min	54.6+/−39.4	15.7+/−18.9	11.2+/−4.2	36.4+/−33.3	29.3+/−28.3
heart	<min	5.2+/−1.0	3.4+/−1.7	2.1+/−0.9	19.5+/−8.7	10.3+/−3.9
lung	<min	5.2+/−2.7	5.9+/−1.5	4.5+/−2.1	118.8+/−60.8	128.0+/−80.4

GT:gene transfer

**Table:**
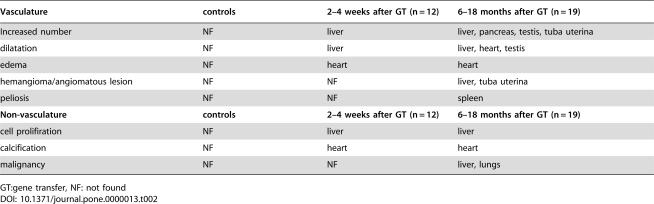
B. Summary of morphological changes in tissues after activation of hVEGF-A165 expression.

Vasculature	controls	2–4 weeks after GT (n = 12)	6–18 months after GT (n = 19)
Increased number	NF	liver	liver, pancreas, testis, tuba uterina
dilatation	NF	liver	liver, heart, testis
edema	NF	heart	heart
hemangioma/angiomatous lesion	NF	NF	liver, tuba uterina
peliosis	NF	NF	spleen
**Non-vasculature**	**controls**	**2–4 weeks after GT (n = 12)**	**6–18 months after GT (n = 19)**
cell prolifiration	NF	liver	liver
calcification	NF	heart	heart
malignancy	NF	NF	liver, lungs

GT:gene transfer, NF: not found

**Figure 4:**
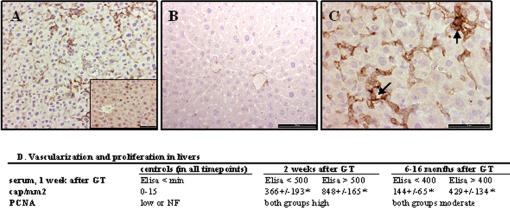
Short term histopathological changes in the liver four weeks after gene transfer. A. Increased number of capillaries showing branching and sprouting (CD 34 immunostaining). Insert: PCNA immunostaining in nuclei correlated positively with the degree of angiogenesis. B. Control liver only revealed weak positivity in hepatic vein branches (CD 34 immunostaining). C. Details of neovascularization in the liver: formation of glomeruloid bodies and enhanced sprouting and branching (pointed with arrows, CD 34 immunostaining). D. Vascularization and proliferation in livers after AdCre gene transfer. ELISA values refer to two subgroups of mice with low and high hVEGF-A_165_ levels in serum. Bar: A insert, C–50 µm; B–100 µm. GT: gene transfer, cap/mm^2^: capillaries/mm^2^, NF: not found, * p<0,05.

Adenoviral gene expression after systemic gene transfer is typically seen in the liver, heart, kidneys, spleen and lungs [27]. Accordingly, changes in histology after hVEGF-A_165_ expression were seen in the liver, heart and spleen in most mice, but usually not in the lungs or in the kidneys ([Table pone-0000013-t001] Table 1). This can be explained by comparison of the human and mouse endogenous VEGF-A protein levels in different tissues. In the lungs the maximal hVEGF-A_165_ protein expression levels were only 2–8% of the mVEGF-A_164_ levels and in the kidneys hVEGF-A_165_ protein expression rapidly decreased to low or undetectable levels. At the early time points, 1–4 weeks after the AdCre gene transfer, histopathological changes were found only in the livers (Figure 4A–C). An increased number of capillaries featured active angiogenesis: formation of glomeruloid bodies, increased sprouting and branching, sac-like structures and focal hemangioma-like structures (Figure 4C). The number of capillaries correlated with the number of proliferating hepatocytes (PCNA positive nuclei) and the expression levels of hVEGF-A_165_ (Figure 4D). Similar results have been seen in transgenic mice which express hVEGF-A_165_ in the liver [18], [29]–[30].

### Long-terms effect of hVEGF-A_165_

**Figure 5:**
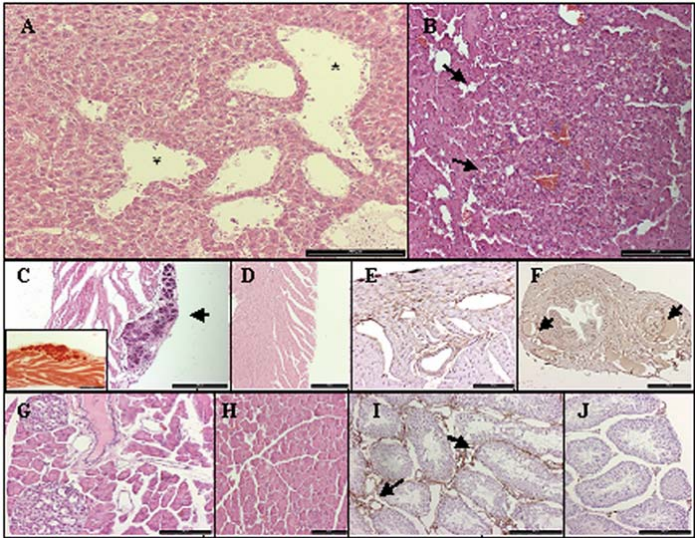
Long-term histopathological changes after activation of hVEGF-A_165_ expression. A. Liver: Dilated hepatic vein branches and mild dilatation of the sinuses (marked with *) (Haematoxylin-eosin). B. Liver: focal basophilic hyperplasia consisting of fat-filled hepatocytes (pointed with arrows), (Haematoxylin-eosin). C. Heart: epicardial calcification in ventricular myocardium (pointed with an arrow), (Haematoxylin-eosin, insert Alizarin RedS staining specific for calcium). D. Control heart. (Haematoxylin-eosin). E. Heart: ventricular myocardium revealed discrete edema and dilatation of subepicardial vessels (CD 31 immunostaining). F. Tuba uterina revealed high vascularization and focally dilated venules and arterioles (pointed with arrows), (CD 31 immunostaining). G. Pancreas with focally increased vasculature (Haematoxylin-eosin). H. Control pancreas. (Haematoxylin-eosin). I. Testes with increased and dilated vasculature (pointed with arrows), (CD 31 immunostaining). J. Control testes. (CD 31 immunostaining). Bar: A, B, C, D, I,J - 200 µm, G, H, E- 100 µm, F- 500 µm.

Within 6–18 months after the AdCre gene transfer increased vascularization and proliferation of hepatocytes persisted in the livers, but morphological abnormalities and signs of active angiogenesis were less common (Figure 5A). All mice were healthy without any major consequences up to 13 months and only minor changes were seen: Out of 19 mice in the long-term follow-up group one mouse had a hepatic focal basophilic hyperplasia of 500 µm in diameter containing cells with a high amount of fat (Figure 5B). In heart, we found discrete edema and in two mice epicardial calcification was present (Figure 5C, E), which can be explained by hVEGF-A_165_ capacity to increase bone formation [31]. In one mouse the fallopian tube was highly vascularized (Figure 5F) and in other mouse increased vascularization, occasional focal fibrosis and paraductal lymphocytic infiltration was found in pancreas. Also, testis of the same mouse was highly vascularized (Figure 5G–J). No changes were found in ovaries and kidneys. Generally, all angiogenic changes were found at the level of capillaries and arterioles/venules and featured increased number of vessels, enlargement of vessels and local angiogenic activity.

**Figure 6:**
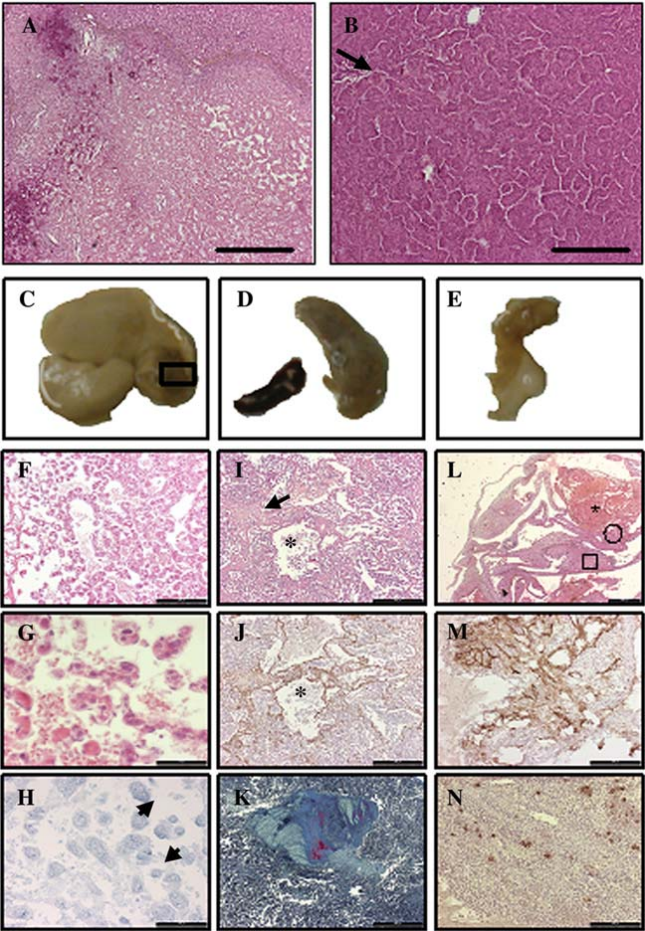
Major changes after 16-months after activation of hVEGF-A_165_ expression. A. Liver: Encapsulated hepatocellular carcinoma was highly necrotic and focally calcified (Haematoxylin-eosin). B. Lung: Papillary adenocarcinoma. (Haematoxylin-eosin). C. Macroscopical changes in liver (box shows the area of the microscopical sections in F–H). D. Macroscopical changes in spleen, control spleen on the left (microscopical sections in I–K). E. Macroscopical changes in paratubarian area (microscopical sections in L–N). F. Cavernous hemangioma featuring focal hyalinization. G. The same tumor mass area with higher magnification (haematoxylin-eosin). H. Weak focal hVEGF-A_165_ immunoreactivity was present in the same area (True Blue as a chromogen), arrows indicate positive cells. I–K. Fibrous scars formed by collagenous and elastic fibres surrounded by dilated cystic spaces, focally filled with blood and revealing inconsistently CD34 positive lining. I. Hematoxylin-eosin. (an arrow points to an area shown in K) J. CD34 immunostaining. K. The same area as in I with higher magnification (stained with Masson Trichrom). Asterix in I and J indicate the same area. L. Cystically dilated paratubarian spaces with signs of old haemorrhage and thrombus formation (marked with *), an open circle points to an area which was highly vascularized as shown in M. (CD31 immunostaining) and a box indicates an area which revealed focal hVEGF-A_165_ immunopositivity shown in N. (hVEGF-A_165_ immunostaining). Bar**:** F, I, J - 500 µm, G, H, K - 100 µm, A, B, M, N - 200 µm L - 1000 µm.

At the eighteen months follow-up after the gene transfer major consequences were seen; one mouse developed a hepatocellular carcinoma in the liver and another had a papillary adenocarcinoma in the lungs (Figure 6A, B). Both of these mice had moderate expression levels of hVEGF-A_165_ in serum after 1 week (240 and 710 pg/ml). We and others have previously seen spontaneous carcinomas in the liver in many mouse models, especially in old female mice with balb/C background [32] and also the transgenic expression of hVEGF-A_165_ especially in the lungs was low (mouse endogenous VEGF-A_164_ levels were 25 times higher than hVEGF-A_165_). Still, we cannot exclude the possibility that hVEGF-A_165_ played a role in the formation of tumors by inducing the growth of dormant tumors or by some other mechanism, especially in the case of hepatocellular carcinoma. In contrast of activated transgenic mice only benign tumors were found in control mice with the same age (wt with Ad gene transfer or transgenic mice without activation); a thecoma in the fallopian tube and an adenoma in the lungs. One hVEGF-A_165_ transgenic mouse died spontaneously 16 months after the gene transfer with abdominal cavity full of blood and serum levels of hVEGF-A_165_ still over 500 pg/ml. Many changes were seen macroscopically in different tissues of this mouse (Figure 6C–E). Microscopically a pendulating cavernous hemangioma (7 millimetres in diameter) was present in the liver with weak focal hVEGF-A_165_ immunoreactivity (Figure 6F–H). Also, the spleen showed a combination of fibrous scars, which were reminiscent of Gandy-Gamna nodules described as a consequence of focal haemorrhage and dilated cystic spaces which were focally filled with blood resembling peliosis (Figure 6I–K). In the same animal the whole paratubary area was highly vascularized and revealed focal hVEGF-A_165_ positivity with signs of old haemorrhage and thrombus formation in dilated cystic paratubary spaces. (Figure 6L–N). All of these findings can be connected with local over-expression of hVEGF-A_165_.

In conclusion, we have created a transgenic mouse model which over-expresses hVEGF-A_165_ after Cre-protein gene transfer. By using different vectors, tissue specific promoters and local gene transfer techniques hVEGF-A_165_ expression can be targeted in desired tissues and cell types in adult mice. In this model the levels of hVEGF-A_165_ expression were moderate and typical angiogenetic changes were mostly found in the liver, but also in some other tissues after systemic gene transfer. Most mice were healthy without any major consequences up to 18 months. However, one mouse died spontaneously because of bleeding into abdominal cavity and having liver hemangioma, haemorrhagic paratubarian cystic lesions and spleen peliosis. Also, two mice developed malignant tumors (hepatocellular carcinoma and lung adenocarcinoma). Thus, we concluded that uncontrolled long-term expression of hVEGF-A_165_ may cause significant pathological changes in target tissues and tight regulation of the transgene expression seems to be a prerequisite for all therapeutic applications aiming at long-term expression of hVEGF-A_165_.

## Methods

### Generation of the Cre controlled human VEGF-A_165_ expression cassette

An oligonucleotide containing restriction sites for Xho I and Sda I was cloned into a Bgl II+Dra II−digested pcDNA3 vector (Invitrogen). The resulting plasmid was named pcDNAmcs. A Sal I and Pst I fragment containing CAG promoter (consisting of the CMV immediate early enhancer and the chicken β–actin promoter) and rabbit β-globin polyA from pCAGGS plasmid (a generious gift from Prof. Jun-ichi Miyazaki) was cloned into XhoI/SdaI cut pcDNAmcs. A loxP site containing an MCS oligo linker was cloned into the EcoRI site of the plasmid. The resulting plasmid was named pCaGGSmcs. A BamHI digested and blunted STOP-cassette (consisting of the SV40 early polyadenylation signal and a splice donor signal [33] from the pBS302 (RIKEN DNA Bank) was incorporated into Pml I site of the pCAGGSmcs. The resulted plasmid was digested with EcoRI and EcoRV and ligated with hVEGF-A_165_ cDNA (digested by EcoRI and EcoRV from pCMV-hVEGF-A_165_). The resulting plasmid was named as pFlox (Figure 1) and first tested in NIH-3T3 cells which were tranduced by Cre adenovirus (containing Cre gene under CMV promoter) at MOI 500. 12 h after transduction, cells were transduced with the pFlox plasmid. Medium samples were taken for human VEGF-A ELISA analysis (R&D Systems, Minneapolis, USA) at different time points.

### Experimental animals

pFlox plasmid was digested with Sal I/Asc I and the resulting fragment was microinjected into the CD2F1 hybrid mice (Balb/C x DBA2). Transgenic mice were analyzed from tail genomic DNA by PCR using specific primers for hVEGF-A_165_ (5′-ccatgaactttctgctgtc-3′ and 5′-tcgtgagattctgccctc-3′) and an internal control gene (ApoB with primers 5′-attgccttagatagtgcc-3′ and 5′-tttgctagatttacacgg-3′), F6 generation mice were used for the experiments. hVEGF-A_165_ transgenic mice (n = 31) and control mice without the transgene (n = 10) were injected via tail vein with recombinant E1-partial E3-deleted first generation serotype 5 Cre adenovirus (1×10^9^ pfu). Another group of transgenic mice (n = 10) were also injected with saline and served as additional controls. A subgroup of animals were sacrificed 1 to 4 weeks after the gene transfer and the rest of the mice were sacrificed 6 to 18 months after the gene transfer. Diet and water were provided ad libitum. Blood samples were taken and circulating hVEGF-A_165_ and endogenous mVEGF-A_164_ levels were measured from tail vein plasma samples at various time points after the gene transfer with enzyme-linked immunoassays (R&D; Quantikine, human VEGF-A and mouse VEGF-A). Routine clinical chemistry assays for aspartyl aminotransferase (ASAT) and C-reactive protein (CRP) were done with Ecoline^R^25 (Merck Diagnostica) and Quickread CRP (Orion Diagnostica kits). During gene transfers animals were anesthetized using s.c. fentanyl-fluanisone (3.15 and 10 mg/kg)/midazolam (5 mg/kg). Mice were sacrificed using carbon dioxide. The arterial tree was perfused with PBS. Tissues samples were collected for the analysis of transgene expression [34] and either post fixed for 2 h (4% paraformaldehyde) and embedded in paraffin or frozen in liquid nitrogen for RNA and protein analyses. All animal experiments were approved by the Animal Care and use Committee the University of Kuopio.

### MRI and ELISA cross-reaction test

Another group of transgenic (n = 8) and control (n = 4) mice were transduced by AdCre or Ad-Tie-Cre or Ad hVEGF-A_165_ (1×10^10^ pfu/ml) in a total volume of 100 µl distributed into the distal hind limb and into the calf of the left hind limb. Right hind limbs served as controls. Only mice which were transduced by AdCre were followed up with MRI, others were used to evaluate possible cross-reaction by ELISA. MRI was performed at 1, 4, 7, 14, 21, 28, and 42 days after the gene transfer. Mice were anesthetized and positioned with straight-tighten hind limbs inside a surface coil. MRI was performed at 9.4 T vertical Oxford magnet (Oxford Instruments, Eynsham, UK) interfaced to a SMIS console (Surrey Medical Imaging Systems, Guilford, UK). Fat saturation was used in all MRI experiments. T_2_-weighted imaging was performed using a spin echo sequence with two adiabatic refocusing pulses. Total echo time (TE) was 44 ms, repetition time (TR) 2000 ms, resolution 256×128, field-of-view (FOV) 22×22 mm^2^ with 4 averages.

### Immunohistochemistry

For immunohistochemical analysis, serial sections (6 µm) were cut and used for stainings. Sections were routinely stained with hematoxylin-eosin and in selected cases with Masson Trichrom, PAS, and Alizarin Red S method for calcium in order to analyze basic changes in different tissues. Tissue vascularization was assessed by immunostaining with CD34 antibody (HyCult biotechnology BV, clone MEC 14.7, dilution 1∶20). Antigen was visualized by avidin-biotin-HRP system (Vectastain ABC kit, Vector Laboratories). Endogenous avidin-biotin activity in liver was blocked by avidin/biotin blocking kit (Avidin/biotin Blocking kit, Vector Laboratories). Additionally, anti-CD 31 rat anti-mouse monoclonal antibody (PECAM-1, BD Pharmingen, clone MEC13.3, 1∶50) and LYVE-1 primary rabbit anti-mouse monoclonal antibody (dilution 1∶1000) were used to assess the vascular system [35]. Both antigens were detected using tyramide signal amplification (TSA Biotin System, PerkinElmer). Hepatocellular proliferation was analyzed by immunostaining for proliferating cell nuclear antigen (PCNA, Neomarkers, clone PC 10, dilution 1∶500). Antigen was detected using the DAKO ARK kit (DakoCytomation Denmark A/S, Glostrup, Denmark). Human VEGF-A (polyclonal goat, R&D Systems, 1∶500) was detected using avidin-biotin-HRP system (Vectastain ABC kit, Vector Laboratories). The signal was visualized with DAB (Zymed, South San Francisco, CA) or True Blue (KPL, Maryland, USA) as a chromogen. ApoTaq kit was used to evaluate apoptosis in livers after the gene transfer. Number of capillaries and apoptosis in the liver sections was counted in five randomly selected fields at 200x magnification using Olympus AX70 microscope [26] (Olympus Optical, Japan) and AnalySIS software (Soft Imaging System).

### RT-PCR and protein extractions

Total RNA was isolated with TRIzol compound (Invitrogen). RNA was DNAse treated and digested before cDNA synthesis with Eco 130I, which cuts the DNA in the middle of the amplification area in order to get rid of possible genomic contamination. cDNA was synthesized from the total RNA with Superscript II (Invitrogen) using random primers. Amplification of the transgene was performed using inner primers for hVEGF-A_165_ as described [36]. Proteins were extracted with T-PER Tissue Protein extraction reagent including Halt™ protease inhibitors and total protein contents were assayed with BCA™ Protein Assay kit (Pierce, USA).

### Statistical analysis

All statistical analysis were done using modified t-test (SPSS 7.5, SPSS Inc). Data are expressed as mean±SD and value of P<0.05 was considered significant.
